# Geochemical constraints on the Hadean environment from mineral fingerprints of prokaryotes

**DOI:** 10.1038/s41598-017-04161-2

**Published:** 2017-06-21

**Authors:** Alexey A. Novoselov, Dailto Silva, Jerusa Schneider, Ximena Celeste Abrevaya, Michael S. Chaffin, Paloma Serrano, Margareth Sugano Navarro, Maria Josiane Conti, Carlos Roberto de Souza Filho

**Affiliations:** 1 University of Campinas, Institute of Geosciences, Campinas, 13083-970 Brazil; 2 University of Concepción, Institute of Applied Economic Geology, Concepción, Casilla 160-C Chile; 3 University of Campinas, School of Civil Engineering, Architecture and Urban Design, Campinas, 13083-889 Brazil; 4 Universidad de Buenos Aires, Facultad de Ciencias Exactas y Naturales, Buenos Aires, C1428EHA Argentina; 5 CONICET-Universidad de Buenos Aires, Instituto de Astronomía y Física del Espacio (IAFE), Buenos Aires, C1428ZAA Argentina; 6 University of Colorado, Boulder, 80302 USA; 7 Alfred Wegener Institute Helmholtz Centre for Polar and Marine Research, Potsdam, 14473 Germany; 8 André Tosello Institute, Campinas, 13087-010 Brazil

## Abstract

The environmental conditions on the Earth before 4 billion years ago are highly uncertain, largely because of the lack of a substantial rock record from this period. During this time interval, known as the Hadean, the young planet transformed from an uninhabited world to the one capable of supporting, and inhabited by the first living cells. These cells formed in a fluid environment they could not at first control, with homeostatic mechanisms developing only later. It is therefore possible that present-day organisms retain some record of the primordial fluid in which the first cells formed. Here we present new data on the elemental compositions and mineral fingerprints of both Bacteria and Archaea, using these data to constrain the environment in which life formed. The cradle solution that produced this elemental signature was saturated in barite, sphene, chalcedony, apatite, and clay minerals. The presence of these minerals, as well as other chemical features, suggests that the cradle environment of life may have been a weathering fluid interacting with dry-land silicate rocks. The specific mineral assemblage provides evidence for a moderate Hadean climate with dry and wet seasons and a lower atmospheric abundance of CO_2_ than is present today.

## Introduction

The geologic record of Earth’s first 500 Myr is confined mainly to Hadean detrital zircons^1,2^. Newly discovered graphite inclusions in some of these zircons extend evidence for the emergence of life to 4.1 Ga^3^. Deep-root models indicate that at ~4.2 Ga Eubacteria and Archaebacteria diverged from the last universal common ancestor (LUCA), which existed in Hadean between ~4.4-4.2 Ga^4,5^. In addition, recent findings^6^ suggest that prokaryotic metabolism likely remained unchanged for billions of years. Hence, the common chemical features imprinted in the metabolic processes of both prokaryotic kingdoms may be inherited from LUCA. This imprint is not limited to organic molecules, such as amino acids, simple sugars, lipids and nucleic acids, but may also include inorganic constituents, i.e. anions, metal ions and neutrally charged solution complexes. These inorganic species likely impacted early biological evolution, and left a signature in the first cellular metabolism^7^. As later organisms evolved and diversified, it is possible they preserved the essential elements of their formation environment in their cytosols^7–13^. This concept, known as “chemistry conservation principle”^11,14^, states that the chemical traits of living cells are more conserved than the changing ambient conditions on Earth. Therefore, chemical similarities among prokaryotes that survived the hypothetical late heavy bombardment at ~3.9 Ga^9^ could provide unique information about the primordial terrestrial environment and complement data from Hadean zircons.

Here we provide a detailed analysis of the inorganic constituents of metabolically and phylogenetically diverse prokaryotes subjected to different culture conditions to unveil mineral fingerprints in their composition and show new evidence for how Hadean Earth conditions may have impacted the origin of life. In the context of rare reports on the elemental composition of mesophilic bacteria (Supplementary Table 1), we analyzed the chemical composition of five species of bacteria (*Acetobacter aceti*, *Alicyclobacillus acidoterrestris*, *Escherichia coli*, *Nesterenkonia lacusekhoensis*, and *Vibrio cholerae*) and two species of halophilic Archaea (*Haloferax volcanii*, and *Natrialba magadii*) (Supplementary Table 2) whose growth requirements vary greatly; i.e. their cultivation temperatures fluctuated between 32 °C and 45 °C, the ambient pH ranged from 3.5 to 10, and the growing media were characterized by distinct chemical compositions (Supplementary Table 3). To determine the impact of culture media composition and cultivation conditions on our results, *E. coli* was grown in the media of *H. volcanii* (*E. coli in HVCM*), *N. lacusekhoensis* (*E. coli in TSB*) and at highly alkaline pH, which is appropriate for the cultivation of *N. magadii* (*E. coli at pH* = *10*). Moreover, *E. coli* was cultivated in a medium prepared with aqueous solution produced from interactions with basalt (*E. coli in BS*).

## Results and Discussion

**Figure 1:**
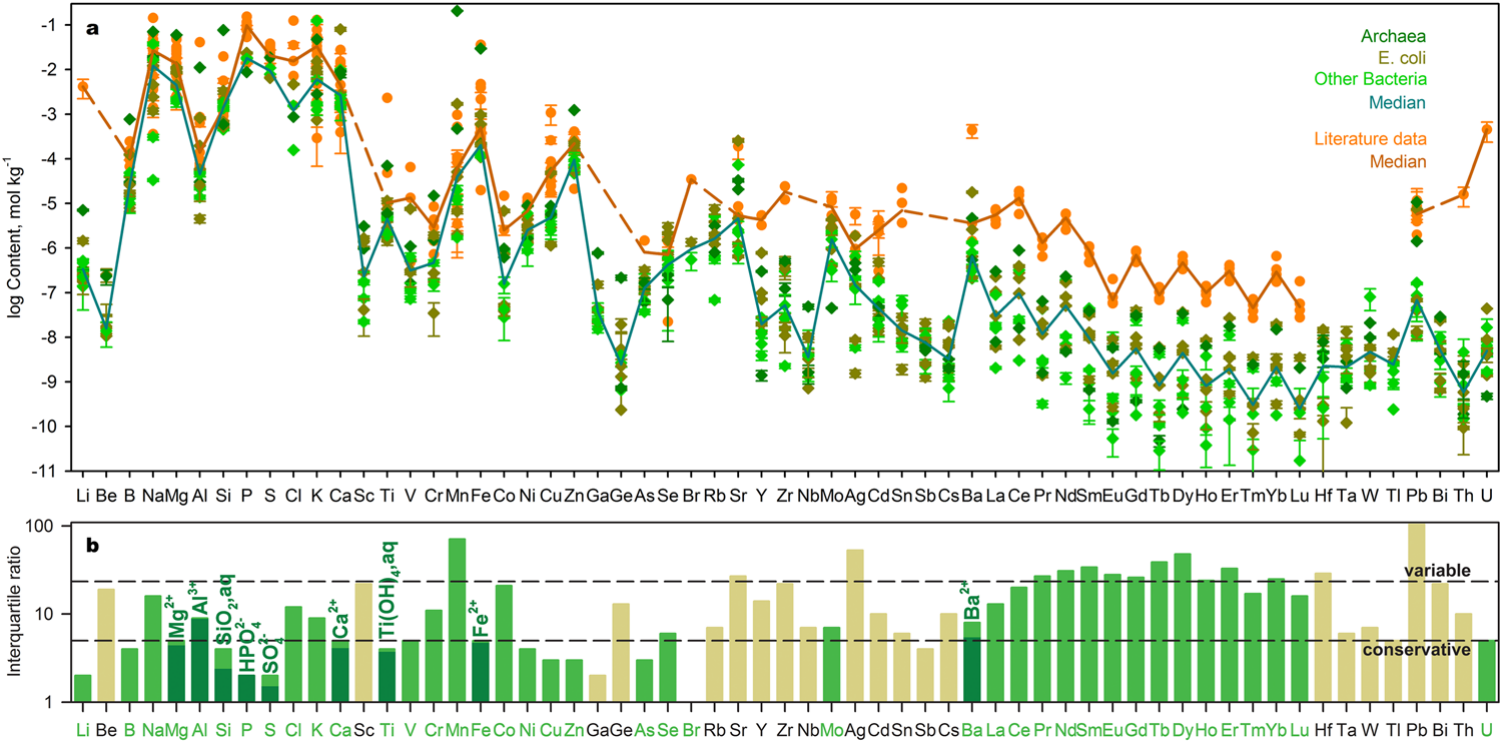
Composition of prokaryotes and dispersion of elemental abundances. (**a**) Observed prokaryotic compositions versus literature data. Uncertainties (1σ) comprise reported values for literature data and variations in our repeated measurements. (**b**) Interquartile ratios calculated for measured elemental contents in prokaryotes (n = 11, see Methods). Biologically essential chemical elements (Supplementary Discussion) are marked by green color. Also the plot illustrates the dispersion of solution species (dark green) supporting the mineral fingerprints considered in this research.

**Figure 2:**
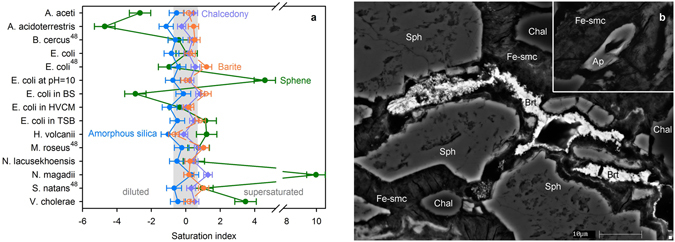
Mineral fingerprints in prokaryotes and minerals precipitated in altered basalts. (**a**) Mineral saturation indices calculated for observed and literature compositions^48^ of various prokaryotes at conditions of their cultivation. For a few species, barite saturation indices (unfilled circles) were estimated using the median valuve of sulfur measured in other species. The shaded region approximates the equilibrium range. Uncertainties are 2σ. (**b**) An analogue for the cradle environment in the modern weathering regolith of Paraná basalts. Mineral abbreviations: Ap – apatite, Brt – barite, Chal – chalcedony, Fe-smc – Fe-smectite, Sph – sphene.

The studied microorganisms show chemical similarities in their inorganic components, possibly inherited from LUCA and its surrounding environment (Fig. 1a). An alternative explanation for this similarity is that the cells incorporated their trace element patterns from growth media with similar trace element signatures. To avoid this possibility, the sets of corresponding pairs of observations on element contents in grown cells and nutrient media were compared using the statistical tests for two related samples (Supplementary Discussion). The implemented tests, as well as direct comparison with compositions of growth media (Supplementary Table 3), provide evidence that prokaryotes tend to control the content of most measured trace elements. In order to accelerate the cultivation of living cells, the standard laboratory cultivation media used in our experiments share similarities with prokaryotes with respect to their main cations, such as Mg^2+^, K^+^ and Ca^2+^. The variation in the elemental composition measured for *E. coli* cultivated at different conditions is comparable with fluctuations among all tested prokaryotes (Fig. 1a, Supplementary Discussion). In fact, growth medium composition, temperature, pH, redox conditions, and the life stage of cells play a role in the chemical makeup of microorganisms^15,16^. Experiments^16^ with *A. violaceus* cultivated in media with differing Mn content, one of the most flexible constituents in living cells, revealed that this element could be accumulated by those bacteria at wide interval (4·10^−5^ - 0.2 mol kg^−1^ of wet weight), which is correlated with medium concentration. However, the consistent growth of *A. violaceus* can continue only at the favorable interval of 4·10^−5^ - 4·10^−4^ mol kg^−1^ of wet weight^16^. All prokaryotes considered here were cultivated in media without enforcing their elemental supply and, hence, observed compositions reveal those favorable intervals. The commonality of the chemical composition of prokaryotes we report here was recently confirmed for species living at extreme hydrothermal conditions^17^, demonstrating that the major trends of prokaryotic inorganic composition are insensitive to environmental settings. Our measurements also agree with the limited measurements available in the literature (Supplementary Table 1). We propose that a few outliers in literature reports shown in Fig. 1a and in Supplementary Table 1 for Li, Ti, Zr, Sn, Ba, Th and U stem from experiments with an excess of those elements in the nutrient media, or due to deprecated analytical techniques. Nevertheless, the bulk mineralization, pH and pe^−^ of prokaryotic cytoplasm can vary with environmental parameters^18^, so that any elemental signature of the cradle environment is best discerned not from the absolute concentrations of elements or solution species, but in ratios of their chemical activities (Fig. 1b). These ratios can be interpreted using approaches traditionally employed in analyzing fluid geochemistry. These methods screen against effects caused by distinct ionic strength and type of speciation in living cells. Changes in the bulk elemental content may differ by factors of 100–1000 while retaining the same activity value of the essential solution species. The chemical composition of any natural fluid is established as a balance between dissolution of primary minerals and precipitation of alteration phases. The precipitating minerals are often in equilibrium with the solution components. To reveal those minerals, we calculated the saturation indices for the extended set of solid phases using both measured cytosolic compositions and literature reports considering Si, Al, Ti, Ba and other key solution components in certain prokaryotic species (Supplementary Table 1). All calculations were performed independently for each species. We found that the fluids corresponding to the composition of each prokaryote are saturated in barite (BaSO_4_), sphene (titanite, CaTiSiO_5_), and chalcedony or amorphous silica (SiO_2_) (Fig. 2a). The saturation index of sphene is pH-sensitive (see Methods). Thus, the saturation indices calculated for species cultivated at strongly acidic or alkaline conditions (*A. aceti*, *A. acidoterrestris*, *E. coli at pH* = *10*, *E. coli in BS*, *N. magadii*, and *V. cholerae*) deviate from equilibrium. This mineral association can precipitate from fluids at wide T-P conditions in various rock-dominated environments with low water-rock ratio. Ti-bearing mineral (sphene) is particularly relevant for determining formation conditions because Ti is often considered as an immobile element and does not migrate for long distances^19^. We found those minerals in the weathering profile of Paraná basalts (22.892764°S, 47.089161°W, Fig. 2b). Recently, the same mineral association was reported for the oldest known basalt paleosols (2.76 Ga) observed in Mount Roe^20^. However, weathering specifics in the Hadean is yet unclear and its geochemical features might be distinct even in Neoarchean regoliths.

**Figure 3:**
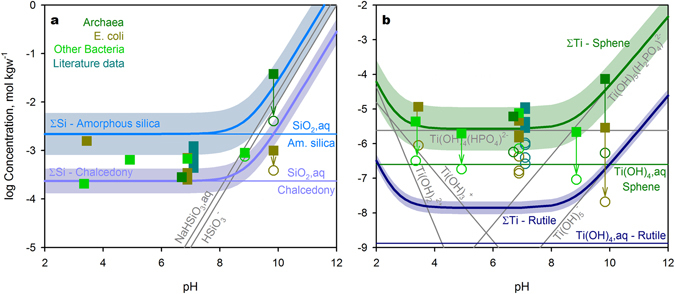
Si and Ti speciation in fluids saturated with respect to various minerals versus contents in prokaryotes. (**a**) The ΣSi and SiO_2_,aq in solutions saturated with amorphous silica and chalcedony. NaHSiO_3_,aq and HSiO_3_^−^ trends correspond to saturation in amorphous silica. (**b**) The bulk Ti content and solution species correspond to saturation level of sphene and rutile at pH = 7 and cation composition of *E. coli*. They are propagated to other pH values assuming the same content of Ti(OH)_4_,aq. In the case of rutile, ΣTi and Ti(OH)_4_,aq are shown only. Uncertainties (2σ) on thermodynamically predicted total Si and Ti are shown by shaded regions. The prokaryotic abundances are marked with squares scaled by their 2σ uncertainty. The unfilled circles reveal the estimated contents of SiO_2_,aq and Ti(OH)_4_,aq in cells. At pH < 8 SiO_2_,aq equals to the bulk Si content.

Mineral fingerprints are encoded in cellular metabolisms as the needs for certain solution species (i.e. Ba^2+^, Ca^2+^, SO_4_^2−^, SiO_2_,aq, Ti(OH)_4_,aq). Ca^2+^ and SO_4_^2−^ are the main cytosolic constituents involved in numerous biochemical processes^21^ (see Supplementary Discussion). Barite crystals can be used as a gravitational sensor^21^ and to control the cell density^22^. Therefore, to maintain its saturation level the product of Ba^2+^ and SO_4_^2−^ contents needs to be constant. SiO_2_,aq or Si(OH)_4_,aq play a role in glycoprotein stabilization^21^. In addition, silica encapsulation is used by bacteria to aid survival under extreme pH conditions^23^. Titanium is a potential reducing agent responsible for the protection of cells from excessive oxidation. It can also participate in photosynthesis and in the fixation of molecular nitrogen^24^. At the stage of prebiotic evolution, SiO_2_,aq and Ti(OH)_4_,aq might catalyze the nucleobase and acyclo nucleoside synthesis^11,25–28^. Under acidic and alkaline conditions (pH < 4 and > 8) the total content of Si and Ti in cytoplasm can be significantly higher than the concentrations of essential solution species involved in metabolic reactions (Fig. 3). This forces the cells to accumulate barren solution species, providing additional energetic consumption and shifts in their chemical makeup.

**Figure 4:**
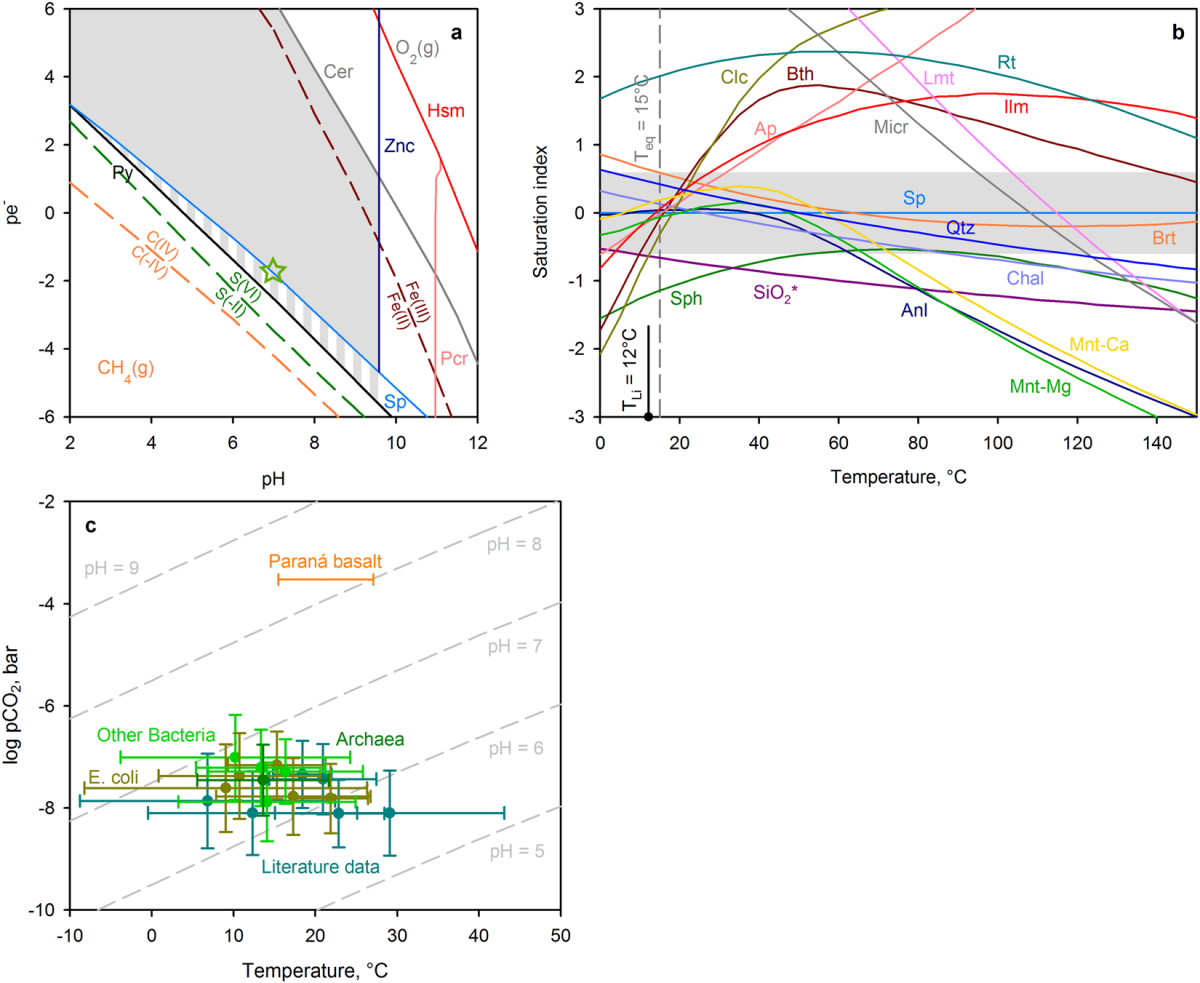
Constraints on the Hadean environment. (**a**) A pe^−^-pH diagram illustrating the redox buffers and stability fields of minerals calculated for *E. coli* composition at 15 °C and 1 bar. The shaded region outlines the conditions where the measured solution is stable. (**b**) Determination of equilibrium temperature (T_eq_) for *E. coli* composition at pH-pe^−^ conditions marked by green star on the Fig. 4a. The grey area represents 2σ uncertainty for equilibrium. (**c**) The upper limit for pCO_2_ in the cradle environment constrained by the precipitation of sphene. Calculations are based on temperatures and pH estimated using the mineral equilibria approach. CO_2_ pressure calculated using compositions of archaea - dark green, *E. coli* – dark yellow, other bacteria - light green, and literature data (Supplementary Table 1) - dark cyan. Uncertainties are 1σ. Mineral abbreviations: Anl – analcime, Ap – Cl-apatite, Brt – barite, Bth – berthierine, Cer – cerianite, Chal – chalcedony, Clc – clinochlore, Fe-smc – Fe smectite, Hsm – hausmannite, Ilm – ilmenite, Lmt – laumontite, Micr – microcline, Mnt-Ca and Mnt-Mg – Ca- and Mg-montmorillonites, Py – pyrite, Pcr – pyrochroite, Qtz – quartz, Rt – rutile, SiO_2_* – amorphous silica, Sp – sphalerite, Sph – sphene, Znc – zincite.

Assuming the obtained prokaryotic compositions sample the composition of natural fluid in which LUCA survived, mineral stability fields and various chemical features of these fluids can be used to estimate the temperature, pH and pe^−^ ranges of the Hadean environment in which life formed. Significant concentrations of redox sensitive trace metals coupled with high iron and sulfur contents impose a lower limit on pe^−^ (Fig. 4a). At values of the reduction potential corresponding to the sulfide-sulfate buffer, As, Cd, Cu, Mo, Pb, Se, V, and Zn cannot be retained in solution and deposit as sulfides, selenides minerals or are captured as impurities by Fe-smectites and pyrite. Because the rare earth elements (REE) patterns do not reveal the well-defined Ce anomaly for all studied prokaryotes (Supplementary Table 2), the upper redox boundary is constrained by precipitation of cerianite (CeO_2_), approximating the ferrous-ferric iron buffer. The precipitation of titanite strongly limits the pCO_2_ in the water-rock system^29^; hence, carbon would have existed in reduced form, i.e. CO, CH_4_ and/or hydrocarbons. This fluid likely interacted with Hadean igneous rocks, whose redox conditions were controlled by the fayalite-magnetite-quartz buffer^2^. Given that the fluid contained reduced C species, igneous magnetite containing ferric iron was probably the main oxidant. To prevent the resulting solution from reduction, the masses of dissolved Fe^3+^ and reduced carbon should be comparable. In order to accomplish this, the water-rock ratio had to be < 1 and the reduction potential variable with time, constrained with stability fields of sulfides and Fe^2+^/Fe^3+^ buffer. A variable reduction potential is easily reached with alternating wet and dry periods: during wet periods the fluids become more reduced promoting the precipitation of sulfides and during droughts the solution becomes oxidized and could remobilize them. The stability fields of Mn minerals and zincite (ZnO) restricts the activity of H^+^ in solution to acidic and neutral values.

Because barite is thermodynamically stable in contact with organic molecules, the upper temperature limit can be established at ~100–140 °C, where thermochemical sulfate reduction by hydrocarbons starts^30^. This upper limit is compatible with the most thermophilic contemporary terrestrial microorganisms, which can grow at temperatures up to 122 °C^31^. A more stringent constraint on temperature can be obtained with the use of empirical geothermometers (see Methods). The Li geothermometer^32^ provides T = 13 ± 6 °C. We can also estimate the temperature and pH of the cradle environment using a mineral equilibrium approach^33^. For the mineral concentrations measured, equilibrium is achieved at 14 ± 9 °C at weakly acidic pH = 6.3 ± 0.3, in good agreement with the Li geothermometer (Fig. 4b,c). The low ambient temperatures suggested by this analysis support the concept of a mesophilic LUCA, as do analyses of ribosomal RNAs and protein sequences^8^. At these parameters, the measured prokaryotic compositions are also in equilibrium with apatite (Ca_5_(OH,Cl)(PO_4_)_3_), montmorillonite ((Ca,Mg)_0.3_Mg_0.6_Al_1.4_Si_4_O_10_(OH)_2_) and berthierine (Fe_2_Al_2_SiO_5_(OH)_4_) – the latter a common Fe-rich clay mineral in rocks exposed to hydrocarbons^34^, which has been found in the Archean Mount Roe basalt paleosols^20^. The metabolic functions of chemical elements linked with those minerals are considered in the Supplementary Discussion.

Prokaryotes share a common ratio in the concentration of Cl and Br, with Cl/Br in *A. acidoterrestris* near 2800 and in *E. coli in HVCM* around 3500. The only literature data available for Cl and Br content shows a Cl/Br ratio of 3600 in phytoplankton^35^. These Cl/Br ratios are well above the oceanic ratio (~650) and the bulk Earth value (420). Solutions or mineral deposits with such high Cl/Br ratios can be formed only in contact with halite (NaCl) or evaporated saline waters^36^.

**Figure 5:**
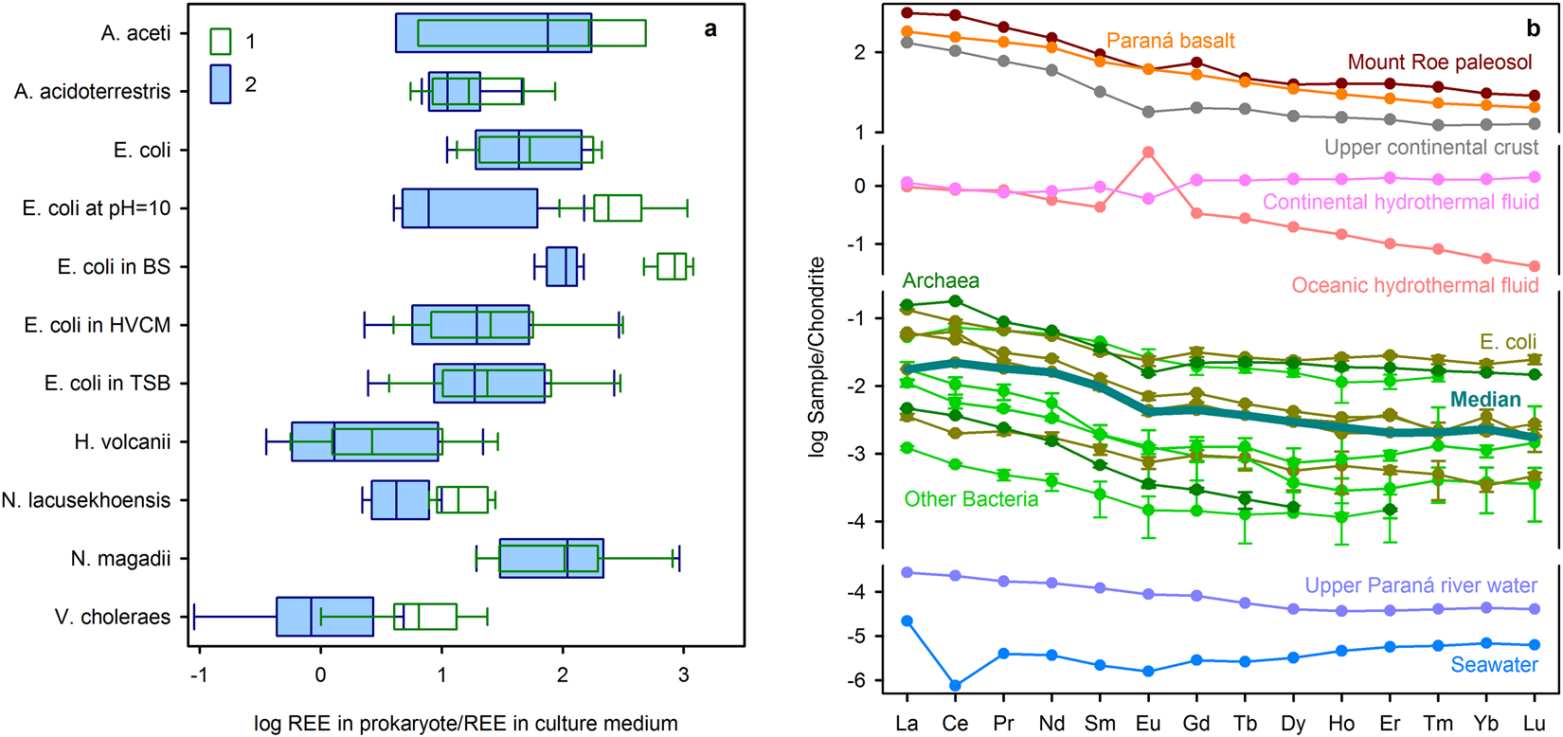
REE in prokaryotes versus (**a**) their contents in nutrient media (1-REE ratios for cells before and 2-after washing procedure) and (**b**) selected rocks and fluids. The C1 normalized plots are shown for Mount Roe basalt paleosol (MR#1^20^), Paraná basalt, upper continental crust^41^, mean trends for continental and oceanic hydrothermal fluids^43^, Upper Paraná river water draining the Paraná basalts^75^ and seawater^76^. The median (dark cyan) is plotted for archaea (dark green), *E. coli* (dark yellow) and other bacteria (light green). Uncertainties are 1σ.

Cytosolic REE concentrations may further constrain the formation environment of LUCA. All tested prokaryotes concentrate REE by up to a factor of 1000 relative to their concentrations in the cultivation media, with individual variations among elements of this group varying by a factor of 100. The only exception is *E. coli in BS*, which initially has a well-fitted pattern in its culture medium (Fig. 5a). Selective accumulation and common REE patterns have been observed for various bacterial species both in laboratory and natural environments, and can be used as a biosignature^37,38^. To accumulate REE, cells use a variety of mechanisms such as surface adsorption, adsorption on extracellular biopolymeric substances and biominerals, and accumulation on carboxylate and phosphate binding sites. However, only a fraction of selectively captured REE can be transported in cytoplasm^39^. Our data support this concept. The patterns of prokaryotes did not change during washing, whereas some species lost up to 87% of initially adsorbed REE (Fig. 5a). There is no significant difference in REE patterns between the bacteria and archaea examined here. This evidences that prokaryotes retain a pattern derived from LUCA. Considering potential sources of the REE signature, the bulk REE contents are higher than in river and sea waters (Fig. 5b). Although Ce anomalies may be negative or positive (0.7–1.6), in all species there is a well-defined negative Eu anomaly (0.5–0.8) typical for weathered rocks^40^. La_N_/Yb_N_ depicts the enrichment of light REE relative to heavy REE with median value 10.7. Those specific patterns resemble the REE distributions in Paraná basalts and especially in Mount Roe paleosols (Table 1). However, the affinity of prokaryotic pattern to the upper continental crust^41^ is also well expressed. By contrast, the characteristic feature of oceanic hydrothermal fluids is a remarkable positive Eu anomaly^42,43^. Hence, the signature of those solutions is dissimilar to living cells. It should be noted that REE compositions of both oceanic and continental hydrothermal fluids are very flexible, depending on the composition of interacting rocks.

**Table 1:** Affinity of REE patterns in prokaryotes to different rock and fluid signatures. The lower value of the parameter corresponds to closer proximity between patterns (see Methods).

REE pattern	Affinity value (1σ)
Upper continental crust^41^	0.31(0.12)
Mount Roe basalt paleosol (MR#1^20^)	0.33(0.11)
Paraná basalt	0.44(0.12)
Upper Paraná river^75^	0.44(0.09)
C1 chondrite and primitive mantle	0.55(0.14)
Continental hydrotherms^43^	0.39–0.85, mean 0.56(0.19)
Seawater^76^	1.10(0.13)
Oceanic hydrotherms^42,43^	0.98–1.97, mean 1.39(0.29)

The set of dissolved source rock minerals required to form a fluid similar to prokaryotic cytosolic compositions can be reconstructed from inverse modeling. To produce this solution, all tested species require the dissolution of rock consisting of Ca-montmorillonite (74–89 vol.%), plagioclase (0–17), ilmenite (6–11), K-feldspar (0–4), magnetite (2–3), goethite (1–3), apatite (0.5–3.7), and pyrite (0.06–0.18). Due to this interaction, the following secondary minerals can precipitate: i.e., Mg-montmorillonite (32–50 vol.%), berthierine (28–40), chalcedony (12–15), sphene (9–13), and barite (0–0.01). Those mineral associations are consistent with the composition of weathering regoliths formed on basalts through interaction with rainwater. The dissolution/precipitation kinetics (see Methods) reveals that the solution coexisted with its mineral surroundings for at least tens of years.

The concept of a mineral fingerprint in the chemical composition of prokaryotic cells is based on the constant activities of certain solution species of Mg^2+^, Al^3+^, SiO_2_,aq, HPO_4_^2−^, SO_4_^2−^, Ca^2+^, Ti(OH)_4_,aq, Fe^2+^, and Ba^2+^ (Fig. 1b). Applied statistical tests (Supplementary Discussion) evidenced that the contents of corresponding chemical elements are highly distinct in the growth media and prokaryotic cytoplasm. Also, we demonstrated on the example of SiO_2_,aq and Ti(OH)_4_,aq that living cells tend to control the content of those solution species, whereas the total contents of Si and Ti vary in wide ranges depending on the pH value in the growth media (Fig. 3). All those components are abundant in cytoplasm and well-known to be essential for biochemical reactions (Supplementary Discussion and references therein). Other considered geochemical features (Li, Cl^−^/Br^−^, REE pattern) are attributed to minor components whose metabolic functions and behavior may be less understood, but they also support the concept.

The commonalities in the chemical composition of prokaryotes presented here suggest that the final stage of prebiotic evolution that preceded the emergence of LUCA (or its early evolution) took place in a dry-land environment. Hence, cavities in surface rocks may have been the first ecological niches to be occupied by early microorganisms^44^. Weathered basalts or basaltic komatiites are the first-order candidates to being those host rocks. They were exposed on the Hadean Earth^45^. However, other rock types bearing clay minerals, minor barite, sphene, silica minerals, apatite and enriched in metals and REE might provide similar fluids. Through modeling and comparison of the results with the chemical and mineralogical makeup of prokaryotes, we suggest that during the Hadean there were climatic conditions with alternating dry and wet periods and moderate ambient temperatures. Atmospheric pCO_2_ must have been below the present atmospheric level (Fig. 4c), and the cradle fluid was enriched in reduced carbon. The weakly acidic pH suggested by this analysis also implies that the atmosphere was not necessarily CH_4_-dominated^46^, but may instead have contained a hydrocarbon haze^47^ that provided C and N-bearing compounds. Alternatively, the cradle environment could have been at a cooling periphery of a dry-land hydrothermal system^11^. However, all other elements necessary for life besides C and N could be mobilized from host rocks. (The works described in references^44,46,47^ approach Archean environments only. There is very little information about the Hadean. Even through the Archean rocks formed during the more recent epoch can provide insights into the Hadean atmosphere and climate, Hadean conditions might differ.)

## Methods

### Cultivation of prokaryotes and samples preparation

*Escherichia coli* (CCT 5050), *Acetobacter aceti* (CCT 2565), *Alicyclobacillus acidoterrestris* (CCT 4384) *Nesterenkonia lacusekhoensis* (CCT 7527) and *Vibrio cholerae* (CCT 7557) strains were obtained from the culture collection of the André Tosello Institute (Campinas, SP, Brazil). *Haloferax volcanii* (DS70) and *Natrialba magadii* (ATCC43099) strains were provided by Dr. X. C. Abrevaya (IAFE – UBA – CONICET, Argentina). All prokaryotes were cultivated in the André Tosello Institute. The major challenge in the observation of chemical composition of bacterial and archaeal cells was to obtain cultures without contaminations from the culture medium or any other substance. Therefore, the washing from the nutrient media was made with ultrapure water^17,48^.

First, unfrozen strains were placed in activation Stock Culture Agar. Then, *E. coli* was transferred to Luria Bertani broth (LB)^49^, *A. acidoterrestris* – to Bacillus Acidocaldarius Medium (BAM)^50^, *N. lacusekhoensis* and *V. cholerae* – to Tryptic Soy Broth (TSB)^51^, and *A. aceti* – to Mannitol medium^52^. BAM was adjusted to pH = 3.5 at 25 °C, the Mannitol medium – to pH = 5, *V. cholerae* was cultivated in medium with pH = 9, and other bacteria – with pH = 7. The active culture incubation proceeded in those media during 72 h. *E. coli*, *V. cholerae*, *A. aceti* and *N. lacusekhoensis* were cultivated at 32 °C with shaking at 130 rpm. *A. acidoterrestris* was incubated at 45 °C without shaking. Haloarchaeal strains were grown in specialized media^53^. *H. volcanii* was grown at 45 °C, aerobically, with shaking at 200 rpm, in Hv-YPC broth^54^ containing (g l^−1^): yeast extract (5), peptone (1), casaminoacids (1), NaCl (144), MgSO_4_.7H_2_O (21), MgCl.6H_2_O (18), KCl (4.2), CaCl_2_ (3 mM), and Tris-HCl (12 mM), with pH adjusted to 7 (at 25 °C). *N. magadii* was grown at 32 °C, aerobically, with shaking at 200 rpm. The growth medium composition^55^ was (g l^−1^): yeast extract (5), NaCl (200), Na_2_CO_3_ (18.5), sodium citrate (3), KCl (2), MgSO_4_.7H_2_O (1), MnCl_2_.4H_2_O (3.6.10^−4^), FeSO_4_.7H_2_O (5.10^−3^), with pH adjusted to 10 (at 25 °C).

We aimed to investigate species dwelling in diverse environments. Hence, the cultivated prokaryotes required chemically distinct growth media. To investigate the impact of growth medium composition on the composition of cells, *E. coli* was grown in the media of other species; i.e., in the culture medium of *H. volcanii* and in the TSB medium of *N. lacusekhoensis*. In the culture media of *A. acidoterrestris* and *N. magadii* the production of *E. coli* was depressed and we could not collect a cell volume adequate for measurements. However, despite the increased growth time (120 h), we cultivated *E. coli* in the LB nutrient medium at pH = 10 attributed to growing conditions of *N. magadii*. *E. coli* was also cultivated in medium prepared with aqueous solution interacted with Paraná basalt (*E. coli in BS*). For this, the pure water acidified with nitric acid to pH = 3.5 was mixed with the basaltic powder in proportion 10 to 1 by weight and heated in an autoclave at 110 °C during 10 days. The elevated temperature and low pH provided a faster extraction of cations. This solution was then filtered and used to prepare the LB medium of *E.coli*. All culture media were sterilized by autoclaving at 120 °C for 20 minutes.

After incubation, cells were separated by centrifugation at 1000 × *g* for 10 min at 10 °C. In order to avoid contamination from the culture medium, collected live cells were suspended in sterilized ultrapure water and centrifuged again, as above. We found that further washing iterations did not impact the chemistry of collected samples. The washing with ultrapure water provided a risk of lysis of cells. Therefore, after each wash procedure, the viability of the cultures was controlled through the hanging drop technique^56^. Experimentally, we found that prokaryotes survived in pure water during ~15 minutes. Therefore, all operations were conducted over that time-frame. All samples were analyzed using the gram stain method to ensure no contamination of the culture by other microorganisms^57^. The optical density of the cultures was measured using a spectrophotometer (Thermo Electron) at λ = 600 nm, and 10 µl aliquots were taken for direct cell counting under the microscope.

The twice washed and centrifuged cultures gave up to 2 g of live biomass. They were collected into PFA vials (Savillex). Weighed portions of ~0.5 g intended for ICP-QMS analyses were separated. Then, 1 ml of distilled HNO_3_ was added and the combination heated on a hot plate at 120 °C until almost complete evaporation of the samples. Next, 0.5 ml of distilled HNO_3_ was added and the heating procedure was repeated. HNO_3_ (100 μl) and ultra pure water (1 ml) were used to take up the residue. The vessels were heated for 5 minutes to obtain a clear solution. The solutions were gravimetrically diluted with ultrapure water to 10 g. All mass losses and gains attributed to evaporation of volatile constituents and addition of ultrapure water and HNO_3_ were taken into account. Therefore, all yielded observations can be considered as the wet weight of the live cells. The rest of samples were used for Karl Fischer titration and TXRF (Total Reflection X-Ray Fluorescence) analyses.

To control for washing losses and gains, all species were sampled 2 or 3 times before washing, after the first and second washings. The culture media before and after the cultivation were also sampled. All samples were run in duplicate. In bulk, 160 samples were analyzed. The revealed uncertainties demonstrate the standard deviation between 4–6 independent observations.

### Analytical methods

Measurements of prokaryotic compositions were performed using an ICP-QMS (Inductively coupled plasma-mass spectrometry with quadrupole mass) equipped with collision cell technology (Xseries^II^, Thermo, Bremen, Germany) in the Isotope Geology Laboratory (IG, Unicamp, Brazil). To provide concentrations in P, S, Cl and Br, TXRF analyses for concentrations of those elements were yielded using the S2 PICOFOX in the Bruker Research Center (Berlin, Germany). The water content in Prokaryotes was observed with Karl Fischer titration in the Institute of Chemistry (Unicamp). Compositions of minerals, elemental maps and BSE images of Paraná basalts were acquired with MEV/EDS Link/Isis Oxford (IG, Unicamp). The bulk chemical composition of basalts was obtained with a Philips PW2404 X-Ray fluorescence (XRF) spectrometer (IG, Unicamp). Also, the types of silica and clay minerals were determined using the EDS/MEV and XploRA (Horiba) Raman spectroscopy in the Institute of Physics (Unicamp). The specific surface areas of minerals were obtained using the BET method with nitrogen gas and the Micromeritics Accelerated Surface Area and Porosimetry (ASAP) 2020 System (Faculty of Chemical Engineering, Unicamp).

### Statistical handling

The obtained compositions of prokaryotes and their growth media were statistically compared using the IBM SPSS Statistics 20 software. We implemented the Wilcoxon Signed Ranks test, the Sign test and the two-sample Kolmogorov-Smirnov test (KS test) (see Supplementary Discussion). The Wilcoxon and Sign tests are used to outline the consistent difference between pairs of observations at the assumption that living cells and nutrient media represent the dependent samples. In contrast, the KS test considers the compositions of growth media and prokaryotes as independent samples and compares their empirical distributions. The rejection of the null hypothesis in all tests provides evidence that the elemental abundances in prokaryotes are distinct from their content in nutrient media. The samples’ size is 11 and *p* is 0.05. We also compared *E. coli* compositions with other prokaryotes using the KS test (*p* = 0.05). The first sample with size of 5 comprised trace element contents in *E. coli* and the second sample with size of 6 in other bacteria and archaea.

The interquartile ratio (IQR) was used to compare the dispersion of abundances of chemical elements in prokaryotes (n = 11) and is calculated as follows:

1 $${\rm{IQR}}={{\rm{Q}}}_{3}/{{\rm{Q}}}_{1}$$

where Q_1_ and Q_3_ are first and third quartiles, respectively. In its turn, the quartiles were estimated for the whole set of IQRs. The chemical elements characterizing by IQR below the first quartile can be characterized by the most stable content in living cells, whereas the highest IQRs exceeding third quartile depict the most variable elements. Also the extent of dispersion was estimated for solution species (i.e. Mg^2+^, Al^3+^, SiO_2_,aq, HPO_4_^2−^, SO_4_^2−^, Ca^2+^, Ti(OH)_4_,aq, Fe^2+^, Ba^2+^) constraining the saturation level of minerals considered in the present research.

### Geochemical calculations

The inverse modeling, speciation calculations and estimation of saturation indices were performed with the software PHREEQC version 3^58^. The uncertainties in equilibrium constants and dissolution/precipitation rates of minerals were calculated using the CRONO software^59,60^. In the course of geochemical simulations to provide reliable results, we applied different databases of thermodynamic parameters. To calculate data shown in Fig. 2a the llnl.dat^61^ was used. This dataset is a part of the standard PHREEQC download. We enforced the database (Database S1) with solubility constants for ilmenite, rutile, and titanite from Supcrt slot07^62^ and with a formation constant for the solution complex Ti(OH)_4_(HPO_4_)^2−^ (ref. 63). The Thermoddem (Thermochemical and Mineralogical Tables for Geochemical Modeling) database^64^ reveals very close values on saturation indices plotted in Fig. 2a. To design Fig 4a,b and for inverse modeling, we used the Thermoddem database because it includes more mineral species than analogical datasets. This tabulation lacks a temperature dependence on solubility constant for chlorapatite. Its values have been calculated using literature data^65^. Ti(OH)_4_(HPO_4_)^2−^ was also added. At the preliminary stage of the research, we utilized the modified pitzer.dat^66^. However, because at the found range of pe^−^ and pCO_2_ the ionic strength of all simulated solutions was less than 1 M, the extended Debye-Hückel model was used to emulate the activities of aqueous species. The kinetic parameters stem from the tabulation composed by Palandri and Kharaka^67^. All considered databases lack the constants for high molecular weight organic species, which are assumed to be abundant in both cytoplasm and in the hypothetical Hadean environment. To outline the possible impact of organics to the speciation model, we implemented a series of additional calculations with different portions of fulvate^2−^ and discussed this issue in details in the Supplementary Discussion.

The deposition of sphene (titanite) from aqueous solution was described with the reaction:

2 $${{\rm{CaTiSiO}}}_{{\rm{5}}}({\rm{sphene}})+2{{\rm{H}}}^{+}+{{\rm{H}}}_{2}{\rm{O}}={{\rm{Ca}}}^{2+}+{{\rm{SiO}}}_{2},\mathrm{aq}+{\rm{Ti}}{({\rm{OH}})}_{4},\mathrm{aq}$$

Its saturation index (SI) is calculating as:

3 $${{\rm{SI}}}_{{\rm{sphene}}}=\,\mathrm{log}({{\rm{a}}}_{\mathrm{Ca2}+}\,{{\rm{a}}}_{\mathrm{SiO2},\mathrm{aq}}\,{{\rm{a}}}_{{\rm{Ti}}({\rm{OH}})4,\mathrm{aq}})+2{\rm{pH}}\,\mbox{--}\,\mathrm{log}\,{{\rm{K}}}_{{\rm{eq}}}$$

where K_eq_ is the equilibrium constant and a_Ca2+_, a_SiO2,aq_, a_Ti(OH)4,aq_ are activities of solution species (mol kgw^−1^ – molal units, kgw means 1 kg of water).

The plots shown in Fig. 3 were calculated in the Microsoft Excel spreadsheets (Database S2) using equilibrium constants at T = 32 °C for Si solution species from Supcrt slot07^62^ and for Ti species from refs 63 and 68. To calculate the concentration of NaHSiO_3_,aq, the content of Na observed in *N. magadii* was used. The concentrations of Ti species were estimated with values for a_Ca2+_, a_SiO2,aq_, a_Ti(OH)4,aq_, a_HPO4–2_ taken the same as predicted for *E. coli*.

The saturation of prokaryotes with respect to sphene (titanite) is an important finding. Unlike rutile, titanite precipitates at low content of HCO_3_^−^ in fluids. Therefore, its presence can be used as an indicator of CO_2_ content in the atmosphere^20,29^. The upper limit for pCO_2_ coexisting with titanite in subaerial sediments can be established with the following equation:

4 $${{\rm{pCO}}}_{2}={{\rm{a}}}_{\mathrm{Ca2}+}{{\rm{a}}}_{\mathrm{SiO2},\mathrm{aq}}/({{{\rm{a}}}_{{\rm{H}}+}}^{{\rm{2}}}{{\rm{K}}}_{{\rm{eq}}})$$

where K_eq_ represents an equilibrium constant for reaction 2CaTiSiO_5_(sphene) + 2H^+^ + CO_2_,g = Ca^2+^  + CaCO_3_(calcite) + 2TiO_2_(rutile) + SiO_2_,aq + SiO_2_(quartz) + H_2_O, and a_Ca2+_, a_SiO2,aq_, a_H+_ are activities of solution species (mol kgw^−1^).

Empirical geothermometers are often used to determine the *in situ* temperature of natural fluids. However, most geothermometers are based on certain contents of Na, Mg, K, Ca^69^. These are important nutrients and their abundance in prokaryotes might be likely considerably modified in the course of biological evolution. According to our observations, the silica (chalcedony) geothermometer^70^ reflects the temperature of cultivation of cells. However, whereas their predictions are rather uncertain, Ca/Na, Mg/Li and Na/Li geothermometers^32,70,71^ suggest low ambient temperatures for the cradle environment. The Li geothermometer^32^ uses the following expression:

5 $$T^\circ {\rm{K}}=2258/(1.44\,\mbox{--}\,\mathrm{log}\,{{\rm{m}}}_{{\rm{Li}}})$$

where m_Li_ is a molal concentration (mol kgw^−1^) of Li in the solution. For *A. aceti*, *E. coli*, *E. coli at pH* = *10*, *E. coli in HVCM*, *E. coli in TSB*, *H. volcanii*, *N. lacusekhoensis*, and *V. cholerae*, the temperature of 13(σ = 6)°C is predicted, for *A. acidoterrestris*, – T = 0 °C, for *E. coli in BS*, – T = 38 °C and for *N. magadii*, – T = 73 °C. Probably the supply on Li in the cells can be modified at extreme pH conditions.

Also the temperature and pH in the cradle environment were reconstructed applying the mineral equilibria approach^33,72^. The technique relies on the use of the composition of a natural fluid to constrain a temperature and pH where a set of alteration minerals are computed to be in equilibrium with the aqueous phase. For all studied prokaryotic species, a series of calculation was yielded at the temperature range of 0.1–150 °C and pH varying between 5 and 9. The redox potential was fixed at the sphalerite saturation level. Pressure was equal to 1 bar at T < 100 °C and corresponds to the saturated steam pressure at T ≥ 100 °C. All minerals tabulated in the Thermoddem database were available for the calculations. The equilibrium temperature was computed as a median of saturation temperatures for the following phases: berthierine, ilmenite, Ca- and Mg-montmorillonite (Database S3). At the same temperature range, compositions of some species also revealed equilibrium with chlorapatite (Ca_5_(PO_4_)_3_Cl), chlorite (Mg_5_Al_2_Si_3_O_10_(OH)_8_) and goethite (FeOOH). When chlorapatite did not fit the equilibrium, the studied fluids were undersaturated with this mineral and supersaturated with hydroxyapatite (Ca_5_(PO_4_)_3_OH). Hence, a solid solution of chlorapatite and hydroxyapatite, like verified in the Paraná basalts and other rocks (Ca_5_(OH,Cl)(PO_4_)_3_), could provide those solution compositions. The composition of *N. magadii* does not provide any equilibrium temperature at the tested range.

In the course of inverse modeling (Database S4), we used the compositions of primary minerals observed in Paraná basalts^73^. Apatite, barite, berthierine, halite, gibbsite, goethite, kaolinite, Ca- and Mg-montmorillonites, pyrite, and sphene were also available in the modeling system. The calculations were yielded at the previously found T-pH-pe^−^ conditions. The uncertainties in solution components were equal to 0.2 that in most cases correspond to 2σ uncertainties specified in the Supplementary Table 2.

The formation time for this solution (Δt_i_) can be calculated as follows:

6 $${{\rm{\Delta }}{\rm{t}}}_{{\rm{i}}}={{\rm{\Delta }}{\rm{x}}}_{{\rm{i}}}/({{\rm{S}}}_{{\rm{i}}}{{\rm{r}}}_{{\rm{i}}})$$

where Δx_i_ represents a dissolved or precipitated amount of mineral species (mol), S_i_ is a surface area of mineral exposed to solution (m^2^) and r_i_ is a dissolution/precipitation rate (mol m^−2^ sec^−1^) calculated at given T, P, pH and pCO_2_ (Database S5). The BET surface areas of apatite, chalcedony, goethite, ilmenite, magnetite, and pyrite correspond to 1 m^2^ g^−1^; K-feldspar, plagioclase and sphene – to 12 m^2^ g^−1^; berthierine and barite – to 30 m^2^ g^−1^; montmorillonites – to 100 m^2^ g^−1^.

Ce anomalies (Ce/Ce*) were calculated here using the expression:

7 $${\rm{Ce}}/{{\rm{Ce}}}^{\ast }=2{{\rm{Ce}}}_{{\rm{N}}}/({{\rm{La}}}_{{\rm{N}}}+{{\rm{\Pr }}}_{{\rm{N}}})$$

where N represents normalization to C1 chondrite. Eu anomalies (Eu/Eu*) were calculated as:

8 $${\rm{Eu}}/{{\rm{Eu}}}^{\ast }=2{{\rm{Eu}}}_{{\rm{N}}}/({{\rm{Sm}}}_{{\rm{N}}}+{{\rm{Gd}}}_{{\rm{N}}})$$

The compositions of C1 chondrite and primitive mantle used here were taken from the tabulation of McDonough & Sun^74^.

The affinity parameter was calculated as a normalized vector distance between all observed prokaryotic REE patterns and a given solution or rock pattern (Database S6). The logarithms of REE contents (C_La_, … C_Lu_, ppm) were considered as coordinates of vector **z**{log C_La_, …log C_Lu_}. They were approximated by a linear trend (y_i_ = a x_i_ + b), which is subtracted from the vector **z**{log C_La_ – a x_La_ – b, …log C_Lu_ – a x_Lu_ – b}, where x_La_ = 1,… x_Lu_ = 14. Based on the yielded normalized vectors **z**_**1**_ and **z**_**2**_, the vector distance (Affinity) was calculated as:

9 $${\rm{Affinity}}=\Vert {{\bf{z}}}_{{\bf{1}}}\,\mbox{--}\,{{\bf{z}}}_{{\bf{2}}}\Vert +\sqrt{{{\sum }_{i}({z}_{1i}-{z}_{2i})}^{2}}$$

where z_i_ = log C_i_ – a x_i_ – b and i = La…Lu.

### Data Availability

External Databases cited in the paper are loaded in the http://www.nature.com/protocolexchange/protocols/4613 and marked with DOI number doi:10.1038/protex.2016.007.

## Electronic supplementary material

Supplementary Discussion

Dataset 1

Dataset 2

Dataset 3
