# Publisher Correction: Geochemical constraints on the Hadean environment from mineral fingerprints of prokaryotes

**DOI:** 10.1038/s41598-018-23130-x

**Published:** 2018-03-14

**Authors:** Alexey A. Novoselov, Dailto Silva, Jerusa Schneider, Ximena Celeste Abrevaya, Michael S. Chaffin, Paloma Serrano, Margareth Sugano Navarro, Maria Josiane Conti, Carlos Roberto de Souza Filho

**Affiliations:** 1 University of Campinas, Institute of Geosciences, Campinas, 13083-970 Brazil; 2 University of Concepción, Institute of Applied Economic Geology, Concepción, Casilla 160-C Chile; 3 University of Campinas, School of Civil Engineering, Architecture and Urban Design, Campinas, 13083-889 Brazil; 4 Universidad de Buenos Aires, Facultad de Ciencias Exactas y Naturales, Buenos Aires, C1428EHA Argentina; 5 CONICET-Universidad de Buenos Aires, Instituto de Astronomía y Física del Espacio (IAFE), Buenos Aires, C1428ZAA Argentina; 6 University of Colorado, Boulder, 80302 USA; 7 Alfred Wegener Institute Helmholtz Centre for Polar and Marine Research, Potsdam, 14473 Germany; 8 André Tosello Institute, Campinas, 13087-010 Brazil

Correction to: *Scientific Reports* 10.1038/s41598-017-04161-2, published online 21 June 2017

This Article contains an error in Equation 9.

$${\rm{Affinity}}={\parallel {\bf{z}}}_{1}\,-\,{{\bf{z}}}_{2}\parallel \,+\,\sqrt{{\sum }_{i}{({{\rm{z}}}_{1{\rm{i}}}-{{\rm{z}}}_{2{\rm{i}}})}^{2}}$$

should read:

$${\rm{Affinity}}={\parallel {\bf{z}}}_{1}\,-\,{{\bf{z}}}_{2}\parallel \,=\,\sqrt{{\sum }_{i}{({{\rm{z}}}_{{\rm{1i}}}-{{\rm{z}}}_{2{\rm{i}}})}^{2}}$$

