# Supplementary material for: Geochemical constraints on the Hadean environment from mineral fingerprints of prokaryotes
Source: Sci Rep. 2017 Jun 21;7:4008. doi: 10.1038/s41598-017-04161-2 (PMC5479841; doi:10.1038/s41598-017-04161-2)
Supplement: Supplementary file 1 — Supplementary Discussion [file 41598_2017_4161_MOESM1_ESM.pdf]

Geochemical constraints on the Hadean environment from mineral fingerprints of prokaryotes

Alexey A. Novoselov, Dailto Silva, Jerusa Schneider, Ximena Celeste Abrevaya, Michael S. Chaffin, Paloma Serrano, Margareth Sugano Navarro, Maria Josiane Conti, and Carlos Roberto de Souza Filho

Supplementary Discussion

1. Comparison of elemental contents in prokaryotes and nutrient media

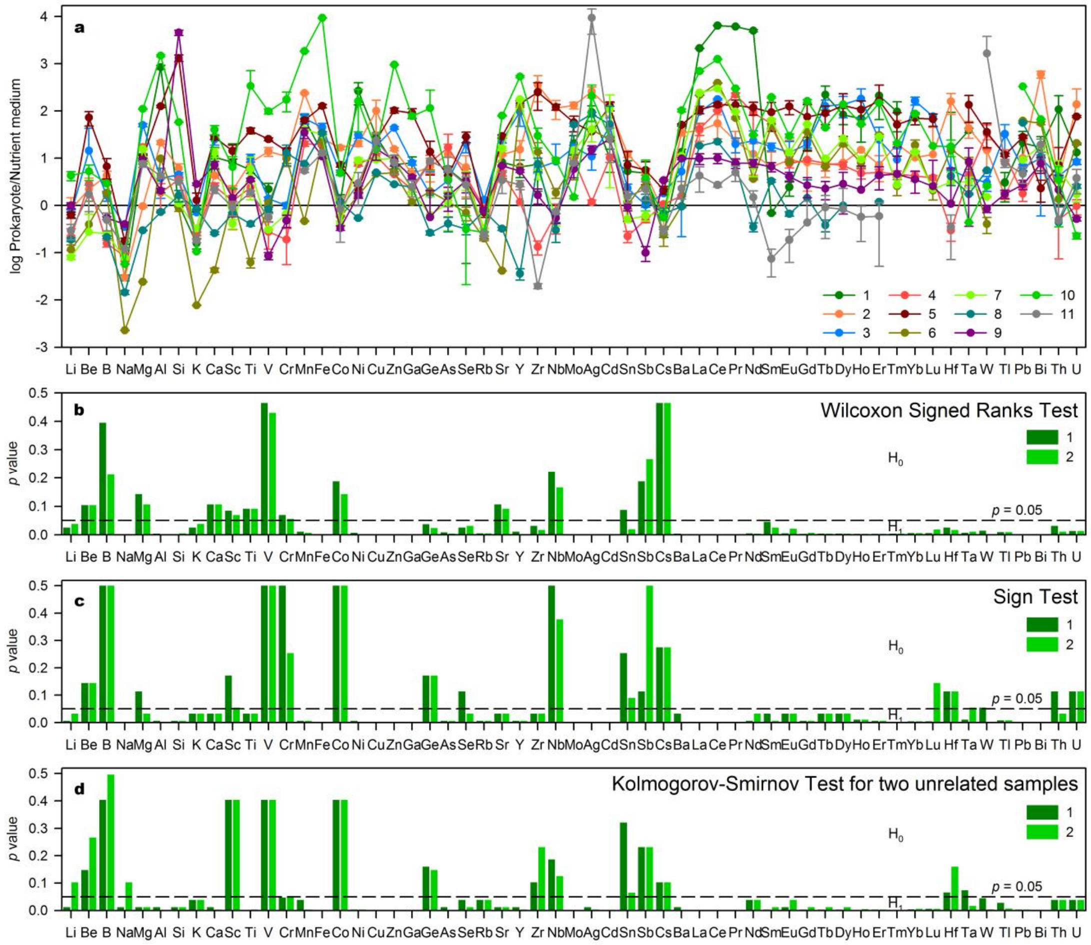

Sup. Fig. 1. Prokaryotic compositions versus their nutrient media. (a) Ratio between concentrations of elements in each prokaryote and in its nutrient medium before cultivation. Prokaryotes: (1) *A. aceti*, (2) *A. acidoterrestris*, (3) *E. coli*, (4) *E. coli at pH=10*, (5) *E. coli in BS*, (6) *E. coli in HVCM*, (7) *E. coli in TSB*, (8) *H. volcanii*, (9) *N. lacusekhoensis*, (10) *N. magadii*, and (11) *V. choleras*. (b-d) Statistical tests comparing observed prokaryotic elemental composition and their nutrient media before (1) and after cultivation (2): (b) Wilcoxon Signed Ranks test, (c) Sign test, and (d) two-sample Kolmogorov-Smirnov test. The null hypothesis (H_0) in all tests supposes that contents of a given element in prokaryotes and nutrient media do not differ and the alternative hypothesis (H_1) – they are distinct. The size of samples is 11. In all cases the null hypothesis was rejected at the critical p value of 0.05.

Prokaryotes tend to adjust their elemental composition (Sup. Fig. 1a). Certain trace elements show values up to 9000 times more concentrated inside the cell than in the nutrient media. In contrast, some other elements show levels by factor of 400 more diluted in the intracellular fluid compared to the extracellular medium. Specifically, the contents of Li and Na in cytosols are below their concentrations in nutrient media.

Three distinct statistical tests (Sup. Fig. 1b-d) reveal close results and indicate that the chemical makeup of living cells is independent on compositions of nutrient media regarding most trace elements. However, the concentrations of

Be, B, Sc, V, Cr, Co, Ge, Nb, Sn, Sb, and Cs resemble the contents in growth media and, probably, prokaryotes do not seek to regulate their supply. Alternatively, their favorable concentrations in cytoplasm may accidentally match the environmental level caused by limited solubility of the considered elements. It should be noted that to accelerate the cultivation of living cells, the laboratory cultivation media employed here are well-fitted according to main cations of prokaryotes, such as Mg, K, and Ca.

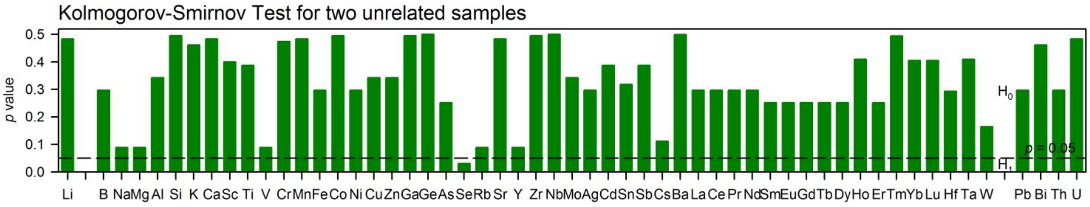

Sup. Fig. 2. Comparison between trace element contents in *E. coli* and other prokaryotes. The null hypothesis (H_0) signifies that two samples belong to the same population and the alternative hypothesis (H_1) to distinct ones. The size of the first sample (*E. coli*) is 5 and the size of the second sample (other bacteria and archaea) is 6. The critical p value is of 0.05.

The Komogorov-Smirnov test showed that there is no difference in variations of trace elements in *E. coli* and other prokaryotes (Sup. Fig. 2).

2. Functions of chemical elements in the cellular metabolism

This brief summary illustrates the fact that many elements may be involved in the metabolic processes, despite their contents within living cells are very low. The functions listed in Sup. Table 1 are only the most representative for each chemical element in prokaryotes. Some elements may be essential for some species and indifferent for others. It must be considered that some elements may be advantageous in case of biotic and abiotic stresses, such as drought, salinity and nutrient toxicity and deficiency[1], regardless their physiological function.

Sup. Table 1. Functions of chemical elements in living cells.

Element	Functions
Li	Interaction with phosphate compounds; i.e. (i) phosphate compounds of the Na^+/K^+ or Mg^{2+}/Ca^{2+} pumps, and (ii) inositol phosphates[2]. Li^+ substitutes Na^+ in the cotransport of amino acids and sugars in various bacteria[3-7]. Li^+ can also replace Na^+ in driving the flagellar motor[8].
B	Stabilization of ribose in RNA[9,10]
Na, K	Na^+/K^+ pump, maintenance of the osmotic pressure[11,12]
Mg	Essential to ATP and nucleic acid chemistry, enzymes and chlorophyll synthesis[13]. Intracellular carbonate benstonite ($(Sr,Ba,Mg,Ca)_6Ca_6Mg(CO_3)_{13}$) is used to control the density and composition of cytoplasm[14].
Al	Required for fluoride activation of G-proteins[15] and participates in the activation of the enzymes of the citric acid cycle[16].
Si	SiO_2,aq or $Si(OH)_4$,aq play a role in glycoprotein stabilization in eukaryotic cells[2]. There have been found prokaryotes capable of utilizing Si, but its function it is not yet clear[17,18]. Also silica encapsulation is used by bacteria to aid survival under extreme pH conditions[19].
P, S	Essential bioelements[13]
Cl	Chloride ion together with Na^+ and K^+ controls osmotic pressure and pH homeostasis[20-22], also used by halogenating enzymes to incorporate into aromatic and aliphatic compounds activated for electrophilic attack[23].
Ca	Participation in many cellular processes including mediation of proteins, activation and stabilization of enzymes, Ca^{2+}/H^+ exchange by ATPase[24-26]. The precipitation/dissolution of intracellular Ca-bearing carbonates (aragonite - $CaCO_3$, benstonite) and sulfates (gypsum – $CaSO_4$, bassanite – $CaSO_4 \cdot 0.5(H_2O)$) can control the density, pH and composition of cytoplasm[14,27]. Also Ca sulfates are used for gravity sensing[27].
Ti	Potential reducing agent responsible for protection of cells from excessive oxidation. It can participate in photosynthesis and in the fixation of molecular nitrogen[16,28].
V	Present in metalloenzymes[29] and involved in nitrogen fixation[2].
Cr	Despite its toxicity, some microorganisms are able to reduce Cr(IV) to Cr(III) as part of their metabolism[30].
Mn, Fe, Co, Ni, Cu, Zn	Present in metalloenzymes[11,29,31-33]. Magnetite (Fe_3O_4) and greigite (Fe_3S_4) are used for magnetotaxis[2,27].
As	As(III) oxidation can be used in anoxygenic photosynthesis[34].

Se	Selenoproteins and Se-dependent enzymes[35].
Br	Br^- is used by halogenating enzymes to incorporate into aromatic and aliphatic compounds activated for electrophilic attack[22]. Also bromide ion incomes in the composition of protein Bromoperoxidase catalyzing the bromination of hydrocarbons[20].
Mo	Present in metalloenzymes[29,36] and involved in nitrogen fixation[2,36].
Ba	Barite ($BaSO_4$) in eukaryotic cells and benstonite crystals are used as a gravitational sensor[2] and to control the cell density[14]. Whereas the functions of barite in prokaryotic cells are not considered, its intracellular precipitation is revealed in various species[37,38]
REE	At low concentrations they stimulate the growth of bacteria[39]. Also REE are considered essential for methanotrophic microbes and used as a cofactor for enzyme methyl dehydrogenase[40].
W	Present in metalloenzymes (oxydoreductases)[29]
U	U(VI) can be used by various groups of bacteria as an electron acceptor[41-46].

3. Impact of organics on the speciation model

High molecular weight organic compounds are abundant in the prokaryotic cytoplasm. However, because the databases of thermodynamic constants consistent with constants for minerals lack the most organic compounds, it was assumed that organic compounds of the prebiotic soup do not impact significantly the solubility of coexisting minerals. In fact, in the frame of the speciation model, the complex organic compounds can affect the solubility of minerals in two ways: (i) they can increase the ionic strength of solution, modifying the activity of charged solution species, and (ii) they can provide other solution species that in its turn can also decrease the activity of species establishing the saturation level of minerals of interest.

Only charged solution species can affect the ionic strength of aqueous solution. At least some part of high molecular organic molecules is charged at physiological pH. DNA and RNA are negatively charged[47,48]. Proteins can be neutral, negatively or positively charged depending on amino acid composition[47,48]. Cell membrane carries negative charge in general[47,48]. However, it is strictly difficult to determine the proportion between distinct organic species and the exact charge which they can produce. In order to constrain the possible impact we implement a series of calculations evidencing the minimal impact of the charge emulated by organics to the saturation indices of minerals considered in this research. We added the charged organic molecules to the system until the ionic strength of solution reached the level of 2 (Sup. Fig. 3). At this value, one elementary charge approximately corresponds to two atoms of organic C. Considering that the significant part of cellular organics is neutrally charged and its high molecular forms, we can conclude that the real value of ionic strength is into the simulated range.

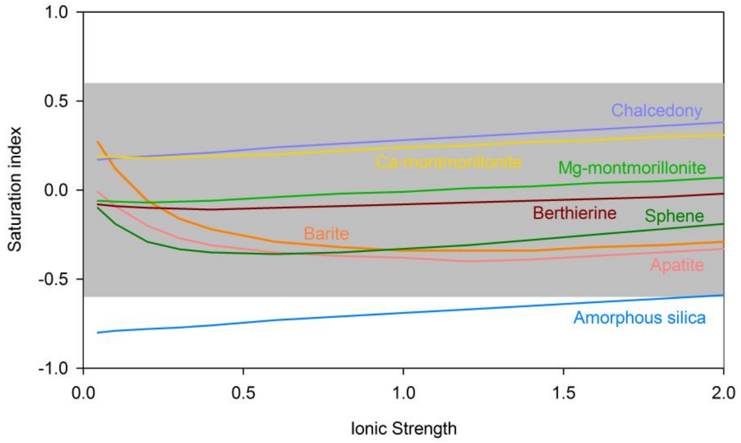

Sup. Fig. 3. Saturation indices of minerals considered in the present research versus ionic strength of solution. The calculations were yielded for *E. coli* composition. The shaded region approximates the equilibrium range (2σ).

The complexation of solution species with organic ligands can potentially decrease the activities of species important for the mineral saturation indices. To avoid this possibility, it is necessary to show that those species are dominant in the cellular solutions and their typical concentrations are near the predicted levels. In this case, the impact of organic ligands can be ignored. Phosphorus is one of the major biogenic elements. In the living cell, phosphorus plays a decisive role as a constituent of phospholipids, nucleic acids and ATP. However, the content of free HPO_4^{2-} is held constant by cellular metabolism at 10-20 mM[49] that is coincident with median value of 9 mM obtained in the present research. This level of phosphate can be established in the system equilibrated with apatite. The robustness of the designed model of speciation is confirmed by the fact that prokaryotes can contain solid magnetite, greigite, barite, silica minerals and others (Sup. Table 1 and references therein). This evidences that the considered minerals and

corresponding solution species (Mg^{2+}, SiO_2,aq, SO_4^{2-}, Ca^{2+}, Fe^{2+}, Ba^{2+}) are at near saturation level in prokaryotic cytoplasm. Also, the prediction of speciation model was independently verified by inverse modeling. If organics would affect the activities of those solution species, their proportions could not provide any realistic mineral association. Our findings on Paraná basalts reveal the predicted minerals existing at the same proportion in natural conditions.

Supplementary references

1. Pilon-Smits, E.A.H., Quinn, C.F., Tapken, W., Malagoli, M. & Schiavon, M. Physiological functions of beneficial elements. *Current Opinion in Plant Biology* **12**(3), 267–274 (2009).
2. Fraústo da Silva, J.J.R. & Williams, R.J.P. The biological chemistry of the elements: The Inorganic Chemistry of Life. 2nd edition, Claredon Press, Oxford, (2001).
3. Chen, C.C., Tsuchiya, T., Yamane, Y., Wood, J.M. & Wilson, T.H. Na+ (Li+)-proline cotransport in *Escherichia coli. J. Membr. Biol.* **84**, 157–164 (1985).
4. Lopilato, J., Tsuchiya, T. & Wilson, T.H. Role of Na+ and Li+ in thiomethylgalactoside transport by the melibiose transport system of *Escherichia coli. J. Bacteriol.* **134**, 147–156 (1978).
5. Tsuchiya, T., Oho, M. & Shiota-Nijya, S. Lithium ion-sugar cotransport via the melibiose transport system in Escherichia coli. *J. Biol. Chem.* **258**, 12765–12767 (1983).
6. Tsuchiya, T., Yamane, Y., Shiota, S. & Kawasaki, T. Cotransport of proline and Li+ in *Escherichia coli. FEBS Lett.* **168**, 327–330 (1984).
7. Uratani, Y., Tsuchiya, T., Akamatsu, Y. & Hoshino, T. Na+ (Li+)/branched-chain amino acid cotransport in *Pseudomonas aeruginosa. J. Membr. Biol.* **107**, 57–62 (1989).
8. Liu, J. Z., Dapice, M. & Khan, S. Ion selectivity of the *Vibrio alginolyticus* flagellar motor. *J. Bacteriol.* **172**, 5236–5244 (1990).
9. Ricardo, A., Carrigan, M.A., Olcott, A.N. & Benner, S.A. Borate minerals stabilize ribose. *Science* **303**, 196 (2004).
10. Grew, E.S., Bada, J.L. & Hazen, R.M. Borate minerals and origin of the RNA world. *Orig Life Evol Biosph* **41**, 307–316 (2011).
11. Mulkidjanian, A.Y., Bychkov, A.Yu., Dibrova, D.V., Galperin, M.Y., & Koonin, E.V. Origin of first cells at terrestrial, anoxic geothermal fields. *PNAS* **109**(14), E821–E830 (2012).
12. Silver, I.A. & Erecinska, M. Energetic demands of the Na+/K+ ATPase in mammalian astrocytes. *Glia* **21**, 35–45 (1997).
13. Mathews, C.K., van Holde, K.E. & Ahern K.G. Biochemistry. Addison-Wesley, San Francisco, Calif., (2000).
14. Couradeau, E., Benzerara, K., Gérard, E., Moreira, D., Bernard, S., Brown Jr., G.E. & López-García, P. An early-branching microbialite cyanobacterium forms intracellular carbonates. *Science* **336**, 459–462 (2012).
15. Nelson, J. Structure and function in cell signalling. Wiley-Blackwell, New York, (2008).
16. Shkolnik, M.Ya. Trace elements in plants. Elsevier, New York, (1984).
17. Das, P. & Das, S. A general classification of silicon utilizing organisms. American Geophysical Union, Fall Meeting 2010, abstract #B51I-046, (2010).
18. Gupta, D. & Das, S. Prokaryotic silicon utilizing microorganisms in the biosphere. American Geophysical Union, Fall Meeting 2012, abstract #B43I-0544, (2012).
19. Hirota, R., Hata, Y., Ikeda, T., Ishida, T. & Kuroda, A. The silicon layer supports acid resistance of Bacillus cereus spores. *Journal of Bacteriology* **192** (1), 111–116 (2010).
20. Sanders, J.W., Leenhouts, K., Burghoorn, J., Brands, J.R., Venema, G. & Kok, J. A chloride-inducible acid resistance mechanism in Lactococcus lactis and its regulation. *Mol. Microbiol.* **27**, 299–310 (1998).
21. Williams, R.J.P. & Fraústo da Silva, J.J.R. The distribution of elements in cells. *Coordination Chemistry Reviews* **200-202**, 247–348 (2000).
22. Anton, J., Oren, A., Benlloch, S., Rodriguez-Valera, F., Amann, R. & Rosello-Mora, R. Salinibacter ruber gen. nov., sp. nov., a novel, extremely halophilic member of the Bacteria from saltern crystallizer ponds. *Int. J. Syst. Evol. Microbiol.* **52**, 485–491 (2002).
23. van Pée, K.H. Enzymatic Chlorination and Bromination. *Methods in Enzymology* **516**, Elsevier, (2012).
24. Yu, X.C. & Margolin, W. Ca2+ mediated GTP-dependent dynamic assembly of bacterial cell division protein FtsZ into asters and polymer networks in vitro. *EMBO J.* **16**, 5455–5463 (1997).
25. Smith, R.J. Calcium and bacteria. *Adv. Microb. Physiol.* **37**, 83–133 (1995).
26. Zakharov, S.D., Li, X., Red'ko, T.P. & Dilley, R.A. Calcium binding to the subunit c of E. coli ATP-synthase and possible functional implications in energy coupling. *J. Bioenerg. Biomembr.* **28**, 483–494 (1996).
27. Raven, J.A. & Knoll, A.H. Non-skeletal biomineralization by eukaryotes: matters of moment and gravity. *Geomicrobiology Journal* **27**(6&7), 572–584 (2010).
28. Udelnova, T.M., Gudina, V.l., Osnotskaya, L.K., Boichenko, E.A. & Tshernogorova, S.M. The content of polyvalent metals in relation to metabolic changes in *Chromatium vinosum*. *Microbiology* **46**, 418–422 (1977).
29. Nitschke, W., McGlynn, S.E., Milner-White, E.J. & Russell, M.J. On the antiquity of metalloenzymes and their substrates in bioenergetics. *Biochimica et Biophysica Acta* **1827**, 871–881 (2013).

30. Cervantes, C.; Campos-García, J., Devars, S., Gutiérrez-Corona, F., Loza-Tavera, H., Torres-Guzmán, J.C., & Moreno-Sánchez, R. Interactions of chromium with microorganisms and plants. *FEMS Microbiology Reviews* **25**, 335–347 (2001).
31. Jakubovics, N.S. & Jenkinson, H.F. Out of the iron age: new insights into the critical role of manganese homeostasis in bacteria. *Microbiology* **147**, 1709–1718 (2001).
32. Mulkidjanian, A.Y. & Galperin, M.Y. On the origin of life in the zinc world. 2. Validation of the hypothesis on the photosynthesizing zinc sulfide edifices as cradles of life on Earth. *Biol Direct* **4**, 27 (2009).
33. Mulkidjanian, A.Y. & Galperin, M.Y. On the abundance of zinc in the evolutionarily old protein domains. *Proc Natl Acad Sci USA* **107**, E137 (2010).
34. Kulp, T.R., Hoeft, S.E., Asao, M., Madigan, M.T., Hollibaugh, J.T., Fisher, J.C., Stolz, J.F., Culbertson, C.W., Miller, L.G. & Oremland, R.S. Arsenic(III) Fuels Anoxygenic Photosynthesis in Hot Spring Biofilms from Mono Lake, California. *Science* **321**, 967– 970 (2008).
35. Hatfield, D.L., Berry, M.J. & Gladyshev, V.N. Selenium. Its molecular biology and role in human health. 3d edition, Springer, New York (2012).
36. Zhang, Y. & Gladyshev, V.N. Molybdoproteomes and evolution of molybdenum utilization. *J Mol Biol.* **379**(4), 881–899 (2008).
37. González-Muñoz, M.T., Fernández-Luque, B., Martínez-Ruiz, F., Chekroun, K.B., Arias, J.M., Rodíguez-Gallego, M., Martínez-Cañamero, M., de Linares, C. & Paytan, A. Precipitation of barite by *Myxococcus xanthus*: Possible implications for the biogeochemical cycle of barium. *Appl Environ Microbiol* **69**, 5722–5725 (2003).
38. Müller, B. Uptake of Barium from vermiculite by the bacterium *Pseudomonas fluorescens* – New indications for barium as a palaeoproxy. *The Open Geology Journal* **8**, 118–123 (2014).
39. Ruming, Z., Yi, L., Zhixiong, X., Ping, S. & Songsheng, Q. A microcalorimetric method for studying the biological effects of La^{3+} on *Escherichia coli*. *Journal of Biochemical and Biophysical Methods* **46**, 1–9 (2000).
40. Pol, A., Barends, T.R.M., Dietl, A., Khadem, A.F., Eygensteyn, J., Jetten, M.S.M. & Op den Camp, H.J.M. Rare earth metals are essential for methanotrophic life in volcanic mudpots. *Environ. Microbiol.* **16**, 255–264 (2014).
41. Lovley, D.R., Phillips, E.J.P., Gorby, Y.A. & Landa, E.R. Microbial reduction of uranium. *Nature* **350**, 413–416 (1991).
42. Lovley, D.R. & Phillips, E.J. Reduction of uranium by Desulfovibrio desulfuricans. *Appl. Environ. Microbiol.* **58**, 850–856 (1992).
43. Francis, A.J., Dodge, C.J., Lu, F., Halada, G.P. & Clayton, C.R. XPS and XANES studies of uranium reduction by Clostridium sp. Environ. *Sci. Technol.* **28**, 636–639 (1994).
44. Shelobolina, E.S., Sullivan, S.A., O'Neill, K.R., Nevin, K.P. & Lovley, D.R. Isolation, characterization, and U(VI)-reducing potential of a facultatively anaerobic, acid-resistant bacterium from low-pH, nitrate- and U(VI)-contaminated subsurface sediment and description of Salmonella subterranea sp. nov. *Appl. Environ. Microbiol.* **70**, 2959–2965 (2004).
45. Wu, Q., Sanford, R.A. & Löffler, F.E. Uranium(VI) reduction by Anaeromyxobacter dehalogenans strain 2CP-C. *Appl. Environ. Microbiol.* **72**, 3608–3614 (2006).
46. Merroun, M.L. & Selenska-Pobell, S. Bacterial interactions with uranium: an environmental perspective. *J. Contam. Hydrol.* **102**, 285–295 (2008).
47. Madigan, M.T., Martinko, J.M., Bender, K.S., Buckley, D.H. & Stahl, D.A. Brock biology of microorganisms. 14th Edition. Pearson, Boston (2015).
48. Alberts, B., Johnson, A., Lewis, J., Raff, M., Roberts, K. & Walter, P. Molecular biology of the cell. 5th Edition. Garland Science, New York (2008).
49. Milo, R. & Phillips, R. Cell Biology by the Numbers. Garland Science, New York (2016).
